# Cancellous allogenic and autologous bone grafting ensure comparable tunnel filling results in two-staged revision ACL surgery

**DOI:** 10.1007/s00402-020-03421-7

**Published:** 2020-04-01

**Authors:** Wolf Christian Prall, T. Kusmenkov, B. Schmidt, J. Fürmetz, F. Haasters, J. H. Naendrup, W. Böcker, S. Shafizadeh, H. O. Mayr, T. R. Pfeiffer

**Affiliations:** 1 FIFA Medical Centre of Excellence, Division of Knee, Hip and Shoulder Surgery, Schoen Clinic Munich Harlaching, Academic Teaching Hospital of the Paracelsus Medical University (PMU), Strubergasse 21, 5020 Salzburg, Austria; 2 Department of General, Trauma and Reconstructive Surgery, Munich University Hospital, Ludwig-Maximilians-University (LMU), Nussbaumstr. 20, 80336 Munich, Germany; 3 Department of Trauma and Orthopaedic Surgery, Cologne Merheim Medical Centre, Witten/Herdecke University, Ostmerheimer Strasse 200, 51109 Cologne, Germany; 4 Department of Orthopaedics and Trauma Surgery, Freiburg University Hospital, Albert-Ludwigs-University, Hugstetter Straße 55, 79106 Freiburg, Germany; 5 Department of Orthopaedic Surgery and Sports Traumatology, Sana Medical Centre Cologne, Aachener Str. 445-449, 50933 Cologne, Germany

**Keywords:** ACL, Anterior cruciate ligament, Two-staged revision, Cancellous bone grafting, Allograft, Autograft

## Abstract

**Objectives:**

Patients with recurrent instability after anterior cruciate ligament (ACL) reconstruction often present with enlarged or misplaced tunnels and bone grafting is required prior to the actual revision reconstruction. Autologous bone grafting features limited quantity and donor site morbidity. These problems may be eliminated utilizing cancellous bone allografts, but their efficiency and reliability have not been investigated systematically. The aim of the present study was to compare tunnel filling rates attained by utilizing either allogenic or autologous cancellous bone grafts.

**Materials and methods:**

A total of 103 consecutive patients were enrolled retrospectively. All patients suffered from recurrent instability and underwent either allogenic or autologous cancellous bone grafting. Computed tomography (CT) was carried out before and after the bone grafting procedure. Based on preoperative CT scans, positioning and maximum diameter of the femoral and tibial tunnels were determined. Tunnel filling rates were calculated as a ratio of pre- and postoperative tunnel volumes. Primary outcome was the tibial tunnel filling rate. Femoral filling rates and density of the grafted bone were assessed secondarily.

**Results:**

Preoperative CT scans revealed no significant differences between the two groups regarding distribution of misplacement and widening of the femoral or tibial tunnel. Postoperative CT scans were conducted after an interval of 5.2 months. Tunnel filling rates of 74.5% (± 14.3) femoral and 85.3% (± 10.3) tibial were achieved in the allogenic compared to 74.3% (± 15.9) femoral and 84.9% (± 9.4) tibial in the autologous group. With *p* values of 0.85 at the femur and 0.83 at the tibia, there were no significant differences between the groups. The density of the grafted bone revealed significantly higher values in the allogenic group.

**Conclusions:**

Utilizing cancellous bone allografts in two-staged revision ACL surgery provides for sufficient and reproducible filling of enlarged or misplaced tunnels. The filling rates are comparable to those achieved with autologous bone grafting. Advantages of allografts are the unrestricted quantity and the absence of any harvesting procedure.

## Introduction

The number of primary anterior cruciate ligament (ACL) reconstructions performed is constantly increasing [1, 2]. A total of over 130,000 procedures are being conducted in the United States annually [3, 4]. While ACL reconstruction reliably restores knee function in the vast majority [5], a relevant percentage of patients suffers a failure emerging as persistent or recurrent instability. The cumulative revision probability constitutes between 4.1 and 6.1% within the first 7 years after the ACL reconstruction [6]. In distinct subgroups, such as young athletes, the mean risk of failure and reinjury is as high as 23% [7]. In specialized institutions revision ACL reconstructions account for more than 10% of all ACL procedures [8, 9]. Persistent or recurrent instability is due to traumatic reinjury, technical errors or lacking graft incorporation [10, 11]. In many cases a combination of all three is evident. A detailed patient history, a thorough clinical examination and profound radiological analyses are absolutely essential when treating revision cases. The primary goal in revision ACL reconstruction is the sufficient fixation of an appropriate tendon graft in good quality bone and anatomically positioned tunnels. Thus, the preexisting tunnels should only be reused in a one-stage procedure if they are anatomically positioned and not critically widened. Unfortunately, patients suffering an ACL reconstruction failure often present with misplaced and/or enlarged tunnels. The misplaced tunnels may converge with the revision tunnels drilled anatomically, potentially jeopardizing sufficient tendon graft fixation. Furthermore, sufficient graft fixation is increasingly difficult to attain in widened tunnels with a diameter of 12 or more millimetres [12, 13]. Consequently, patients featuring misplaced and/or critically widened tunnels often require a two-staged revision. The first step procedure covers removal of tendon graft remnants and interfering implants, milling of the sclerotic tunnel walls as well as cancellous bone grafting. Once sufficient void filling and proper bone graft incorporation are accounted for, the second step procedure comprises the actual revision ACL reconstruction. The cancellous bone grafted during the first step procedure is thereby predominately harvested at the patients’ iliac crest [14–19]. But the harvesting procedure itself features significant disadvantages: it increases the surgical effort, goes along with pain and other morbidities at the harvesting site [20] and the quantity of cancellous bone is limited [21].

With regard to these disadvantages, the utilization of allogenic cancellous bone offers an appealing alternative. The shortfall of the harvesting procedure and the basically unlimited quantity constitute obvious advantages. While the utilization of autologous cancellous bone grafts derived from the iliac crest has been investigated and tunnel filling rates between 78 and 83% have been reported [18], no study has investigated the utilization of allogenic cancellous bone grafts. Therefore, it is unknown whether allogenic cancellous bone grafting ensures tunnel filling rates comparable to those achieved with autologous cancellous bone derived from the iliac crest.

The aim of the present study was to compare tunnel filling rates attained by bone grafting utilizing either allogenic or autologous cancellous bone. It was hypothesized that cancellous bone allografting and autografting would show equivalent tunnel filling rates, femoral and tibial. Furthermore, it was hypothesized that the bone density of the tunnel filling measured in Hounsfield units would be significantly higher in cancellous bone allografting compared to autografting. Primary endpoint was the tibial tunnel filling rate as revealed by computed tomography (CT) prior to the second step procedure.

## Materials and methods

The hospitals’ databases of two study centres were searched for two-staged ACL revision reconstructions between January 2016 and December 2018. During this period one study centre routinely utilized allograft and the other autograft. Inclusion criteria were failure of the ACL graft due to traumatic or non-traumatic reason, the existence of CT scans before and after the bone grafting procedure as well as the utilization of either allogenic cancellous bone or autologous cancellous bone derived from the patient´s iliac crest. Patients, that did not present again after the bone grafting procedure, featured incomplete radiological data sets or underwent simultaneous additional procedures (e.g. high tibial osteotomy) were excluded. The demographic data were collected from the patients’ records. The following parameters were collected: patient age, gender distribution, duration of the void filling procedure and duration of hospitalisation as well as the interval between filling procedure and postoperative CT examination. The study was carried out according to the Declaration of Helsinki. The ethic committee of the University of Munich approved the study under the running ID number 18-114. All patients gave their written consent to participate in this study.

### CT analyses of the preexisting tunnels

CT analyses of the preexisting tunnels with regard to placement and maximum width were conducted in all cases as described before [22]. The CT scan datasets were exported to OsiriX DICOM Viewer V8.0 and processed into 3D surface models. The femoral tunnel position was analysed after digitally subtracting the medial femoral condyle and using a true mediolateral view. The tibial tunnel position was assessed after digitally subtracting the femur and using a craniocaudal view right-angled to the tibial plateau. The centre of the femoral tunnel aperture was determined according to the grid method [23–25]. Accordingly, the centre of the tibial tunnel aperture was determined in a rectangular measurement frame drawn over the craniocaudal view of the proximal tibia [22, 25, 26]. The frame is tangent to the posterior articular margins of both medial and lateral tibial condyles, to the most anterior articular margin of the medial tibial condyle and to the medial and lateral margins of the articular surface. Values are given as percentage of the total distance from deep-to-shallow and from high-to-low for the femoral tunnel aperture as well as from anterior-to-posterior and from medial-to-lateral for the tibial tunnel aperture. For the purpose of this study the ideal aperture centres were defined as follows: 24.8% deep-to-shallow and 28.5% high-to-low at the femur as well as 43.3% anterior-to-posterior and 47% medial-to-lateral at the tibia [22]. The distance between the actual and the ideal aperture centre was determined as percentage point values. Tunnel positioning was considered anatomical when the centre of the aperture was inside a 10 percentage point margin around the ideal insertion point [22, 26]. The maximum femoral and tibial tunnel diameters were determined measuring the largest width at right angles to the tunnel axis on the sagittal, coronal or axial planes. The milling of the sclerotic walls was anticipated in these measurements. In cases of double bundle tunnels and for the purpose of this study, the distance between both tunnel apertures was cut in half to define the centre of an arithmetical tunnel aperture and the largest diameter of both tunnels was taken.

### Bone grafting procedure

All patients underwent general or spinal anaesthesia and were placed in a supine position. The operations were conducted with the leg being positioned in a mobile leg holder. The bone grafting procedure was conducted as published previously [22]. Exsanguination was generated and a tourniquet was inflated to 250 mmHg. The arthroscopic portals and the open approach to the distal entry of the tibial tunnel were reestablished. Meniscal lesions were treated, and chondral lesions were shaven and debrided whenever indicated. Remains of the ACL graft were removed wherever present and both tunnels were identified. All metal ware or bioresorbable materials present were removed. All soft tissues inside the tunnels were debrided and special care was taken to entirely remove the sclerotic walls, either by introducing cannulated drill bits with gradual increasing head diameters or by utilizing an arthroscopic burr. Once visualisation of both tunnels revealed healthy cancellous bone at the entire circumferences, the arthroscopically assisted bone grafting procedure was carried out using cones and push rods. Both tunnels were retrogradely filled with bone graft, repeatedly compressing the cancellous bone mass. A rasp was introduced via the anteromedial portal ensuring temporally closure of the aperture of the tibial tunnel and avoiding trespassing of bone graft into the knee joint. Once sufficient filling of both tunnels was achieved, instruments were removed, and wound closure was conducted. In the allogenic bone grafting group peracetic acid sterilized freeze-dried cancellous bone chips purchased from the German Institute for cell and tissue replacement (DIZG Deutsches Institut für Zell- und Gewebeersatz, Berlin) were utilized.

In the autologous bone grafting group, the harvesting procedure was conducted simultaneously with the arthroscopic procedure. A separate incision was established over the iliac crest. The incision was deepened through the fascia and the abdominal muscles were partially dissected off. Two cylindric spongiosa bone blocks were harvested based on the extent of the reamed tibial and femoral tunnel using disposable cutting tube sets (OATS^®^ Arthrex, Naples, USA). Additionally, cancellous bone was harvested with bone curettes. The bone blocks were inserted in the tunnels using a press-fit technique. Thereafter, the fascia was reconstructed, and wound closure was carried out. After the bone grafting procedure, the patients were mobilised on crutches for 2 weeks and then admonished to bear full weight but to avoid cutting and pivoting stress.

### CT analyses after the bone grafting procedure

The postoperative CT scans were conducted after an interval of at least 3 months. The tunnel filling rates were determined as described before [22, 27]. Again, CT data sets were exported to an OsiriX DICOM Viewer and further analysed. In brief, the tunnel volumes prior to the bone grafting procedure (*V*1) were determined. Manual segmentations of the femoral and the tibial tunnel were carried out in the axial view and over the entire tunnel lengths. The tunnel outlines were marked manually on every single slice using the pencil tool to define the region of interest (ROI). Based on the ROI, the tunnel volume was calculated. Thereafter, the tunnel volumes after the bone grafting procedure (*V*2) were recalculated after setting a threshold of Hounsfield units (HU). A threshold of 200 and 100 HU was set for the femoral and the tibial tunnel, respectively [22, 28]. The filling rate was calculated as percentage ((*V*2/*V*1) × 100). Furthermore, the mean density of the grafted bone was determined in HU.

### Statistical analyses

The tibial tunnel filling rate was the primary outcome parameter. No data exist on minimal clinically important differences of filling rates in two-staged revision ACL surgery. For the purpose of this study, a difference of 10% between the two groups was considered to have the potential of becoming clinically significant. From the literature the mean tibial filling rates utilizing autologous cancellous bone harvested from the iliac crest was 78 with a SD of 14 [18]. Under these conditions and using the G*Power software Version 3.1, the sample size was calculated to be 86, with the probability of less than 5% for type I error and a power of 95%. Thus, with a total of 52 in the allogenic and 51 in the autologous group an adequate statistical power was ensured.

Metric results are given as mean values with standard deviations. Statistical analyses were performed using unpaired *t* tests for parametric and Mann–Whitney *U* tests for non-parametric datasets. The Chi-square test was used for nominal results. Statistical analyses were conducted with the SPSS software version 10.0; the level of significance was set at *p* ≤ 0.05.

## Results

**Fig. 1:**
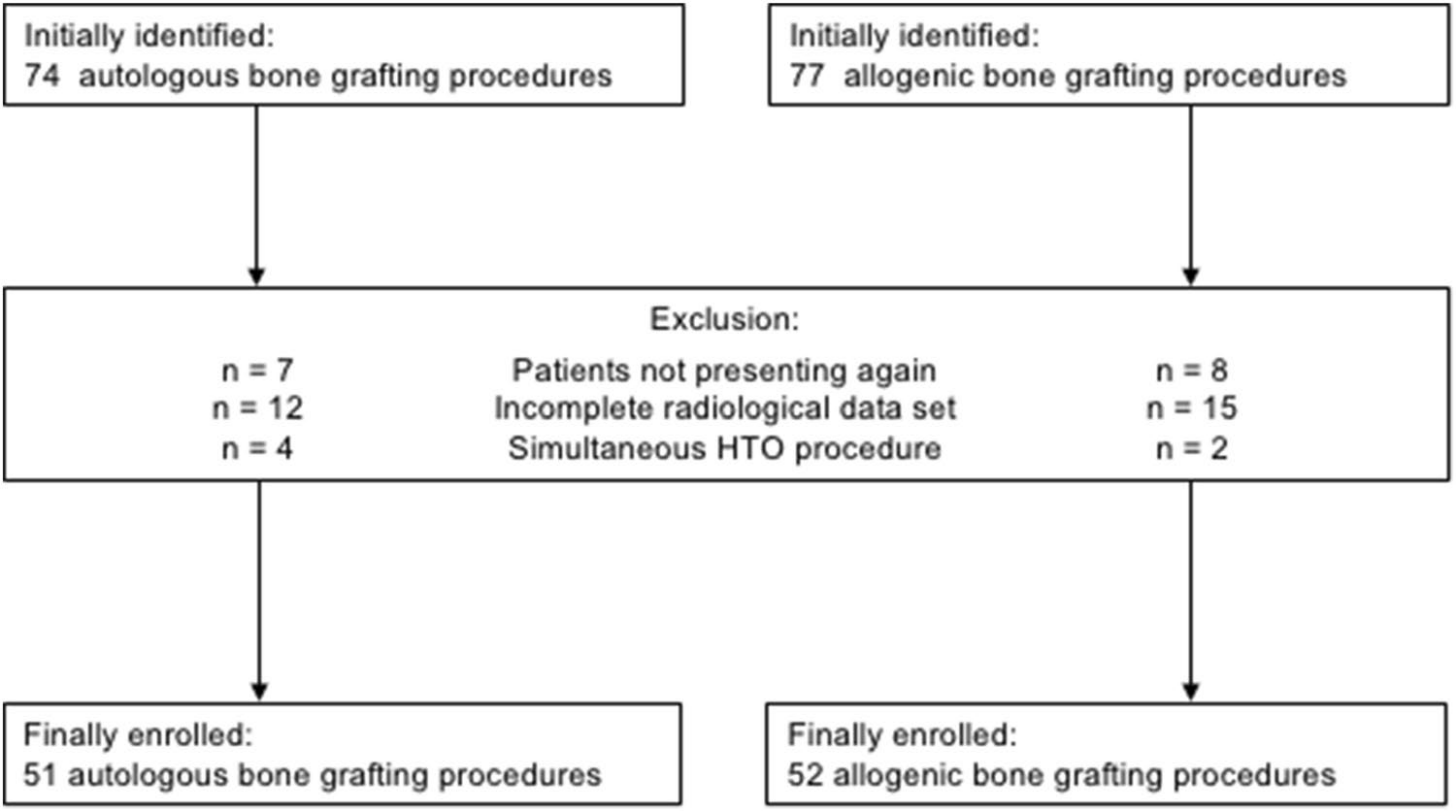
Flow chart illustrating the numbers of initially identified, excluded and finally enrolled patients. *HTO* high tibial osteotomy

A total of 103 consecutive patients were enrolled in this study (Fig. 1). In all cases the indication for a two-staged procedure was given due to misplaced or critically widened tunnels as revealed by preoperative CT analyses. The operations had been carried out by five senior surgeons of the group (W.C.P., J.F., S.S., H.O.M and T.R.P.). The patients’ demographic data are presented in Table 1. Between both groups, there were no significant differences with regard to the duration of the bone grafting procedure as well as the interval between the bone grafting procedure and the postoperative CT scan. In the allogenic group, the patients were significantly younger, and the duration of the hospitalisation was significantly shorter.

**Table 1:** Patients’ demographics

	Allogenic	Autologous	*p* value
Patients (*n*)	52	51	
Age (years)	31.8 (SD 9.5)	27.4 (SD 8.4)	0.01
Gender distribution (m:f)	33:19	33:18	0.9
OP (min)	86.2 (SD 21.4)	90.9 (SD 24.7)	0.31
Hospitalization (days)	2.0 (SD 1.1)	4.0 (SD 1.2)	0.0001
Bone grafting—CT (m)	5.1 (SD 2.7)	5.3 (SD 4.1)	0.73

There were no significant differences regarding the gender distribution, the duration of the filling procedure and the interval between the filling procedure and the postoperative CT. Across the groups, significant differences were revealed in terms of age and the duration of hospitalisation

**Fig. 2:**
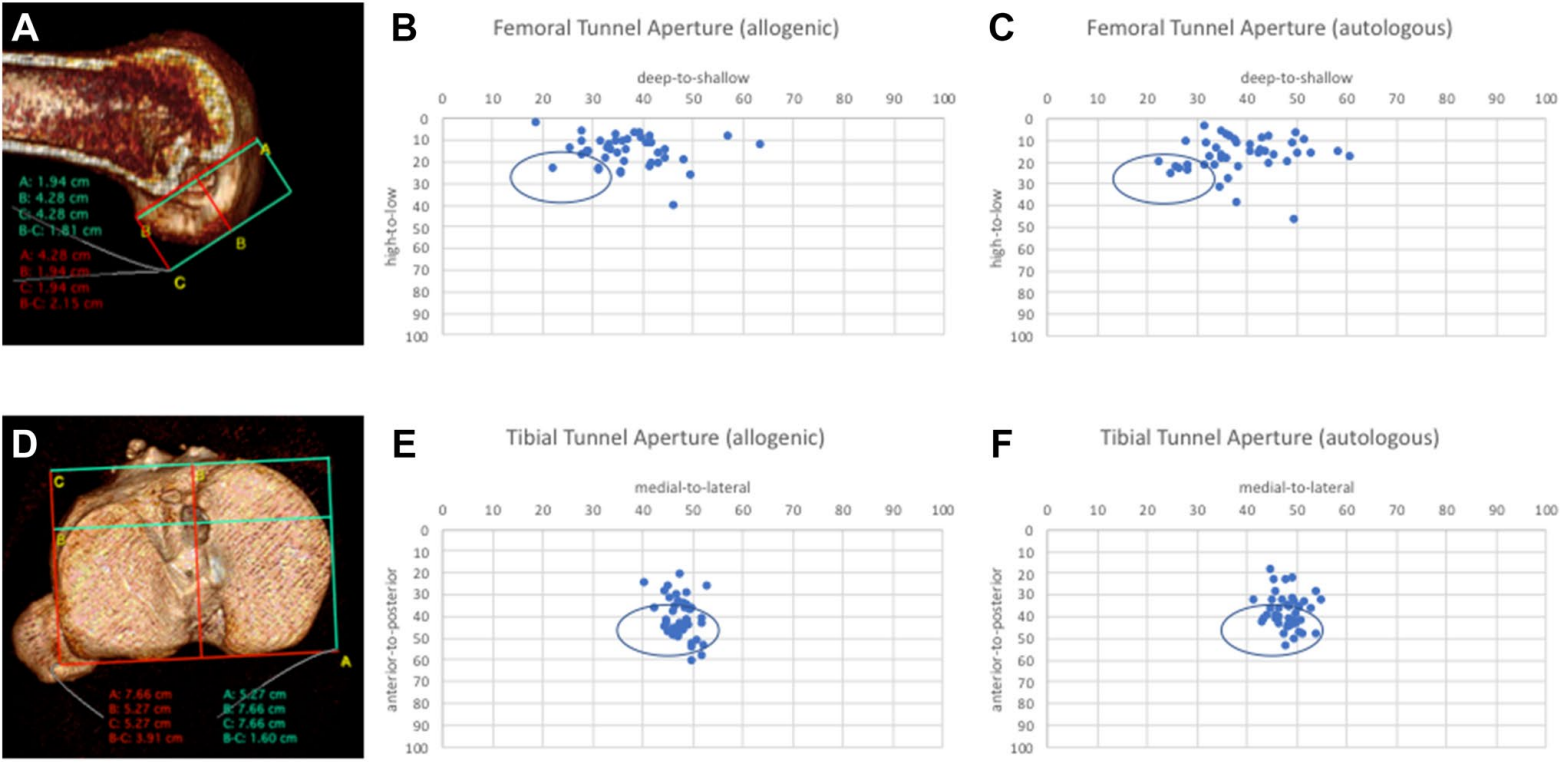
3D CT analyses of the preexisting tunnels (**a**, **d**) revealed that the vast majority of femoral tunnels were positioned to high and to shallow in the allogenic (**b**) and the autologous bone grafting group (**c**). Malpositioning of the tibial tunnels was less evident in the allogenic (**e**) and the autologous group (**f**). No significant differences were detected between the two groups

The preoperative CT analyses revealed a femoral tunnel misplacement in the vast majority of the patients across both groups. Only three and seven femoral tunnel apertures fulfilled the definition of anatomical positioning in the allogenic and the autologous bone grafting group, respectively. All the other tunnels were positioned too high and/or too shallow. With a mean of the 20.7 percentage points (SD 7.2) in the allogenic and 20.6 percentage points (SD 8.4) in the autologous group for the distance between the actual and the ideal centre of the femoral tunnel aperture, there was no significant differences between the two groups (*p* = 0.97). With regards to the tibial tunnel positioning, 35 and 33 of the apertures fulfilled the criteria of anatomical placement in the allogenic and the autologous group, respectively. All the other tunnels were predominately positioned too anterior. With a mean of the 9.4 percentage points (SD 9.6) in the allogenic and 8.5 percentage points (SD 5.4) in the autologous group for the distance between the actual and the ideal centre of the tibial tunnel aperture, there was no significant differences between the two groups (*p* = 0.55). The distribution of tunnel positioning is presented in Fig. 2.

**Fig. 3:**
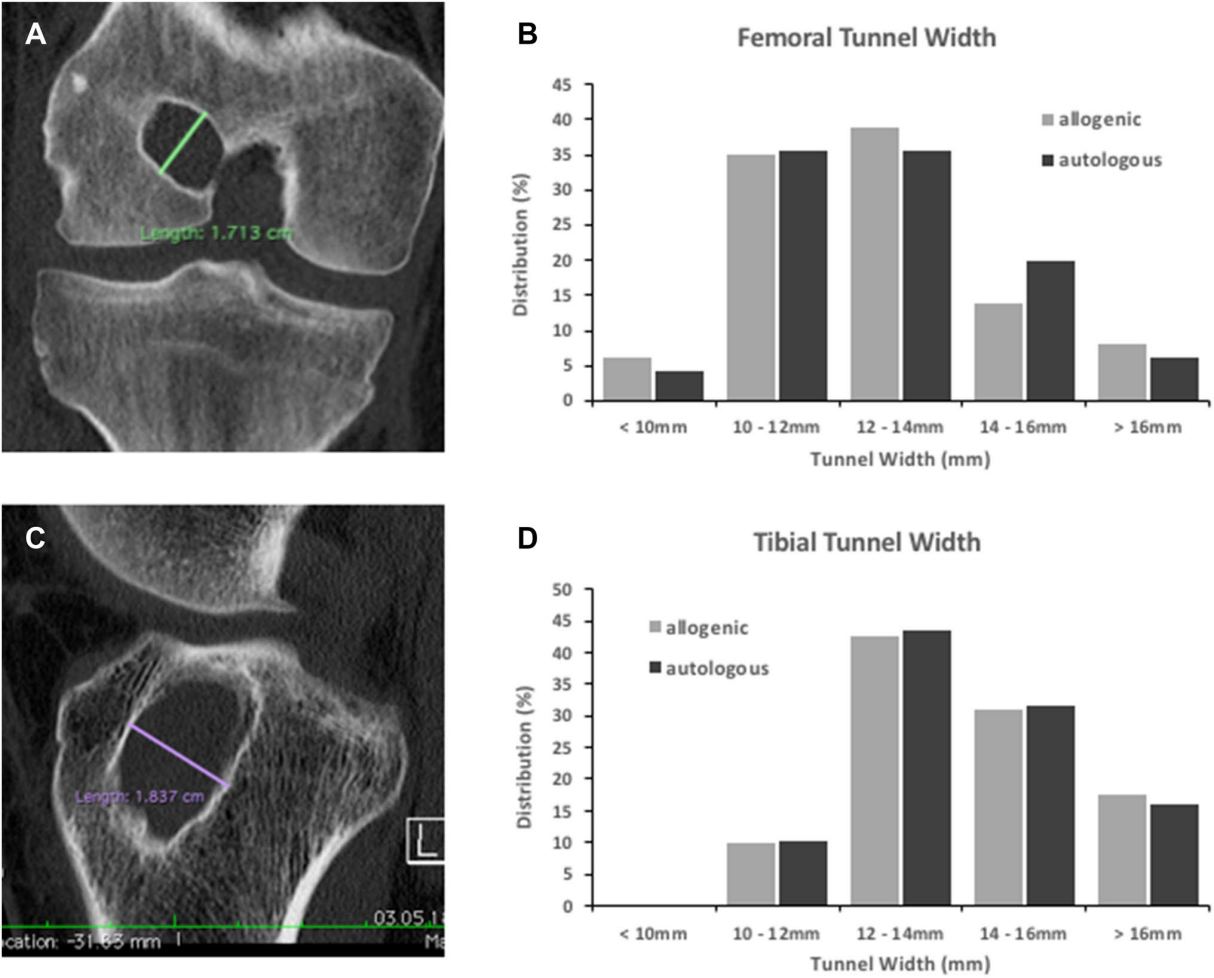
CT analyses of the preexisting tunnel widths. The maximum tunnel diameters were determined perpendicular to the femoral (**a**) and the tibial tunnel axis (**c**). No significant differences concerning the distributions of femoral (**b**) and tibial tunnel widths (**d**) were evident between the two groups

The preoperative CT analyses further revealed a femoral mean tunnel width of 12.4 mm (SD 2.1) in the allogenic and 12.8 mm (SD 1.8) in the autologous group. The mean tibial tunnel widths were 14.3 mm (SD 1.8) and 14.0 mm (SD 1.7) in the allogenic and the autologous group, respectively. With *p* values of 0.25 and 0.43, there were no significant differences for the femoral and the tibial tunnel width between the two groups. The distributions of the tunnel widths are presented in Fig. 3.

**Fig. 4:**
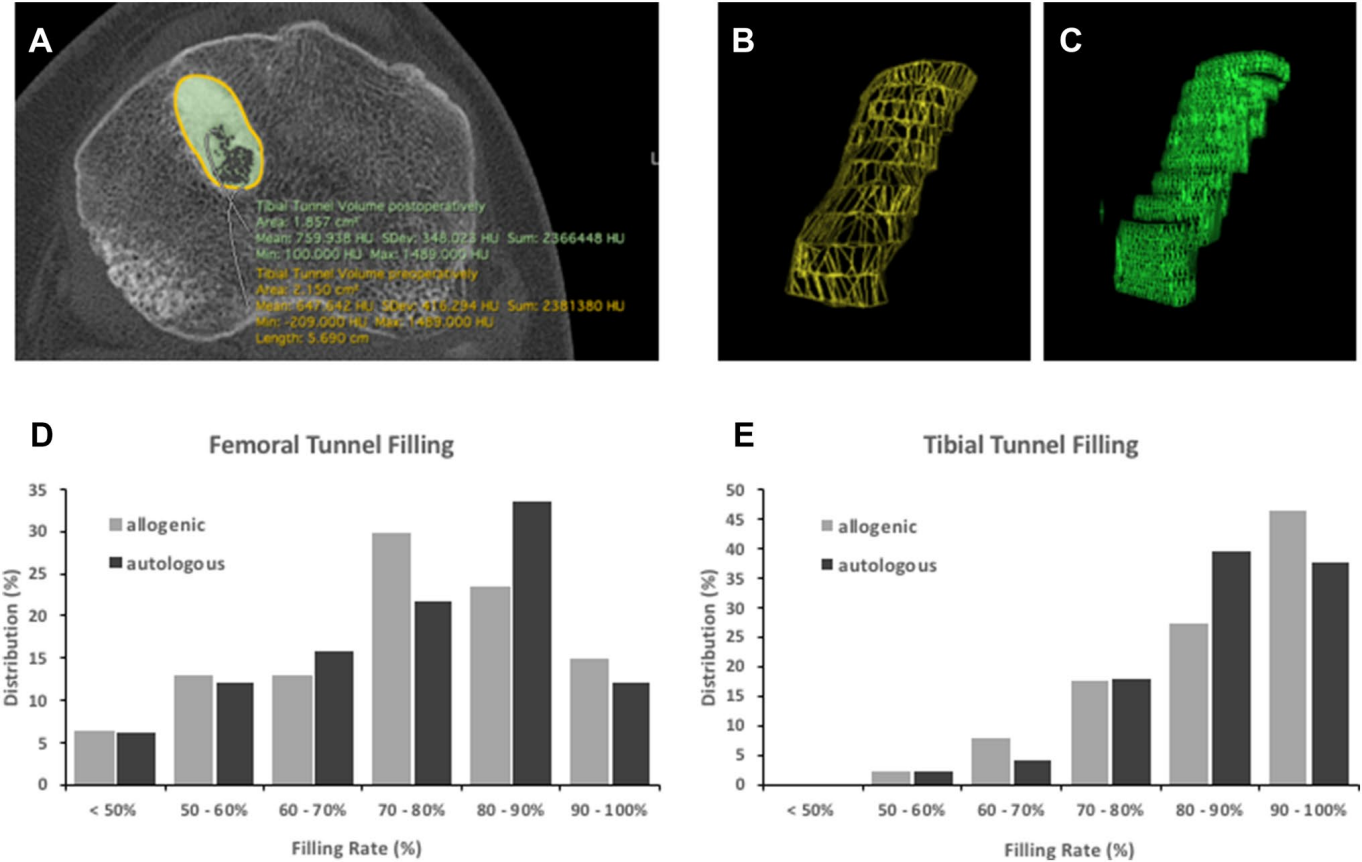
The tunnel filling rates were determined as ratio of the pre- and postoperative tunnel volumes (**a**–**c**). Comparable mean filling rates of the femoral and the tibial tunnels were achieved by allogenic and autologous cancellous bone grafting. The graphs illustrate the distribution of the femoral (**d**) and tibial tunnel filling rates (**e**)

The determination of the tunnel volume before and after the bone grafting procedure revealed mean femoral filling rates of 74.5% (SD 14.3) and 74.3% (SD 15.3) in the allogenic and the autologous group, respectively. With a *p* value of 0.85, there was no significant difference between the two groups. At the tibia, the mean tunnel filling rates were 84.3% (SD 10.3) and 84.9% (SD 9.4) in the allogenic and the autologous group, respectively. With a *p* value of 0.83, there was no significant difference between the two groups. The distribution of the tunnel filling results is presented in Fig. 4. The analyses of the density of the grafted bone revealed mean density values of 559.2 HU (SD 140.8) and 513.7 HU (SD 109.6) in the femoral and the tibial tunnels of the allogenic group. In the autologous group, mean density values of 435.2 HU (SD 109.0) and 435.9 HU (SD 96.6) were detected in the femoral and the tibial tunnel, respectively. With *p* values of < 0.001 in both cases, the mean grafted bone density was significantly higher in the femoral and the tibial tunnels of the allogenic group.

## Discussion

The present study reveals that utilizing allogenic and autologous cancellous bone grafts in two-staged revision ACL surgery provides sufficient void filling of both the femoral and tibial tunnel. Both bone grafts ensure these filling rates in a highly reproduceable manner and no significant differences could be revealed comparing the two groups. This is the first study providing detailed results on filling rates of misplaced and enlarged tunnels in two-staged ACL revision surgery systematically comparing the utilization of allogenic and autologous cancellous bone.

Looking into the patients’ demographic, surprisingly a significant difference between the two groups was noted regarding the mean age (*p* = 0.01). No plausible explanation could be found to account for this difference. Nevertheless, with a mean age of 31.8 in the allogenic and 27.4 years in the autologous group, the values are well in line with mean ages reported in other studies on ACL revision surgery (range 25.5–35.4 years) [8–10, 16–19, 22, 27, 29, 30]. Furthermore, almost two third of the patients in both groups were male. A higher amount of male patients in studies on ACL revision surgery is coherently reported across the literature [8, 9, 11, 16, 29–31].

The extended analyses of the preexisting tunnels of all 103 patients included revealed a tunnel malpositioning in 90.3% at the femur and in 34% at the tibia. All malpositioning at the femur had been conducted too high and/or too shallow. Regarding the upper limits of tunnel widening, values differ between 10 and 15 mm in the literature [32]. For the purpose of this study, a widening of 12 mm and more was defined critical. A critical tunnel widening was evident in 60.2% at the femur and in 90.3% at the tibia. These findings indicate that it is not a single but rather a combination of conditions that underly the indication for a two-staged procedure in the majority of patients. The association of ligament malposition and tunnel widening is a phenomenon also observed in other studies [16, 18, 22, 33]. Concerning the extend of tunnel malpositioning and the distribution of tunnel widenings, no differences were noted between the two groups (Figs. 2, 3).

A significant difference was revealed concerning the duration of hospitalisation (*p* < 0.001). After the bone grafting procedure, patients of the autologous groups featured a significantly longer inpatient treatment compared to the allogenic group. Although no major complication of the harvesting procedure including infection, haematoma or pelvic fracture occurred in the present cohort, the mean difference was more than two days. One possible explanation could be the pain due to the harvesting procedure. It has repeatedly been reported, that bone grafting at the iliac crest can account for severe and prolonged pain [20, 34–36]. Besides that, the harvesting of autologous bone is an additional procedure which may extend the duration of the entire operation. Nevertheless, the present study only revealed an insignificant difference of less than five minutes between the two groups. The underlying cause is supposedly the engagement of a second surgeon, who harvested the bone simultaneously to the ongoing preparation of the tunnels in most of the cases. To date, patients that require a two-staged ACL revision surgery predominately undergo a void filling with cancellous bone harvested at the patients’ iliac crest [14–19]. However, the harvesting procedure increases the surgical effort, is associated with relevant morbidities [20] and can only provide a limited quantity of cancellous bone [21]. Against this backdrop, the utilization of allogenic cancellous bone offers an appealing alternative. The shortfall of any harvesting procedure and the unlimited quantity of cancellous bone may improve the patients’ comfort and allow for reproducible as well as sufficient tunnel filling rates. In the present study only peracetic acid sterilized freeze-dried cancellous bone was used for allografting. The transplantation of peracetic acid sterilized and avital allogeneic bone tissue is associated with a minimal risk of bacterial, viral and non-viral transmission and does not trigger any clinically significant immune reactions in contrast to other allogenic bone grafts [37, 38].

The present study is the first systematic investigation on the utilization of allogenic cancellous bone grafts in the context of two-staged ACL revision surgery. The analyses of 3D CT scans before and after the tunnel filling procedure revealed comparable results in the allogenic and the autologous group. At the femur, filling rates of 74.5 and 74.3 were detected (*p* = 0.85), respectively. The femoral filling rates basically match the results achieved in other studies utilizing autologous cancellous bone from the iliac crest (83%) and the ipsilateral femur (74.7–76.1%), or silicate-substituted calcium phosphate (88%) [18, 22, 27, 32]. The differences between these studies may either be explained by actually different results or by differences in the data acquisition and analysis protocols. The primary outcome parameter of the present study was the tibial filling rate (Fig. 4). Here, no significant differences between the two groups could be revealed (*p* = 0.83). With mean filling rates of 85.3 and 84.9% in the allogenic and the autologous group, the results are comparable to those detected in other studies. In general, detailed knowledge on filling rates is limited. In a recently published systematic review of bone graft options in two-stage revision ACL only two of the included studies provided data on filling rates [32]. Van Recum et al. achieved femoral filling rates of 78 and 86% utilizing autologous bone graft from the iliac crest and silicate-substituted calcium phosphate, respectively [18]. Grafting of autologous cancellous bone harvested at the ipsilateral femur resulted in filling rates of 87.4 and 94% [22, 27]. Uchida et al. grafted bone cylinders derived from the iliac crest and achieved filling rates of 93.8% in predefined sections of the preexisting tunnels [19]. The present study not only reveals the equivalent sufficiency of allogenic and autologous cancellous bone grafting in terms of tibial filling rates, it further demonstrates the high reproducibility of both procedures as shown by the low standard deviations. However, striking and highly significant differences were revealed when comparing the mineral density of both bone grafts (*p* < 0.001). At the femur, a mean 559.2 and 435.2 HU was detected for allogenic and autologous cancellous bone grafts, respectively. At the tibia, mean values of 513.7 and 435.9 HU were measured across the groups. Tie et al. determined a mean physiological density of 211.7 at the distal femur and 104.9 HU at the proximal tibia [28]. Nevertheless, higher mean density values secondary to tunnel filling procedures have repetitively been reported. According to the values of the present study, von Recum et al. revealed mean values of 406–420 HU after grafting autologous cancellous bone derived from the iliac crest [18]. The grafting of autologous cancellous bone harvested by a reamer–irrigator–aspirator at the ipsilateral femur averaged 574.0 HU for the femur and 516.7 HU for the tibia [22]. Furthermore, void filling utilizing silicate-substituted calcium phosphate resulted in mean density values as high as 1095 and 1121 HU [18]. Thus, physiological density values seem to be exceeded by any grafting procedure. Furthermore, the extend of exceedance seems to depend on the amount of calcified matrix as well as the operative technique especially with regards to the graft compaction.

Limitations of the study encompass general shortcomings of retrospective cohort studies as well as the constrained clinical predictions that may be deduced from the present radiological findings. Allogenic and autologous cancellous bone grafting comparably ensure sufficient tunnel filling rates in the CT scans conducted 5.1–5.3 months postoperatively. A time period of 4–6 months was set as the standard minimum across the studies evaluated in the recent systematic review by Salem et al. [32]. Nevertheless, the bone grafting procedures only lay the foundation for the subsequently conducted ACL revision reconstruction. Whether both grafting procedures also allow for comparable anatomical revision tunnel positioning and comparable clinical outcomes has to be investigated in future studies.

## Conclusion

The utilization of allogenic cancellous bone grafts in staged ACL revision surgery provides for sufficient and reproducible filling of enlarged or misplaced tunnels. The filling rates are comparable to those achieved by grafting of autologous cancellous bone harvested at the patients’ iliac crest. Advantages of allografts are the unrestricted quantity and the shortfall of any harvesting procedure. Whether cancellous bone allografting also ensures for accurate revision ACL reconstruction and comparable functional outcomes should be investigated in future studies.
